# Facial morphology analysis of children with non-syndromic cleft lip and palate in a local population

**DOI:** 10.12669/pjms.40.5.8485

**Published:** 2024

**Authors:** Rabia Anjum, Saqib Mahmood, AH Nagi, Saima Chaudhry

**Affiliations:** 1 Rabia Anjum, BDS, MPhil. Assistant Professor, Dept of Oral Pathology, University of Health Sciences Lahore, Pakistan; 2 Saqib Mehmood, MBBS, MSc, PhD. Retd. Professor, Dept of Human Genetics and Molecular Biology, University of Health Sciences Lahore, Pakistan; 3 AH Nagi, MBBS, FCPS, PhD. Retd. Professor of Pathology, Dept of Morbid Anatomy and Histopathology, University of Health Sciences Lahore, Pakistan; 4 Saima Chaudhry, BDS, MPhil, PhD. Professor of Oral Pathology, Director CHPL, The University of Lahore, Lahore, Punjab Pakistan. Adjunct Faculty University of Health Sciences, Lahore, Pakistan

**Keywords:** Cleft lip, Cleft palate, Facial Measurements, Photographs

## Abstract

**Objectives:**

To study the facial morphology in children with non-syndromic cleft lip and palate by applying numerical facial analysis on photographs for planning and evaluating treatment outcomes.

**Methods:**

This descriptive study was conducted from March 2020 to July 2020 in the Department of Oral Pathology, University of Health Sciences and Cleft Lip and Palate Hospital, Lahore Pakistan. A total of 104 patients of both genders with an age range from three months to thirteen years were included. Photographs of the participants were taken to measure facial anthropometrical landmarks including facial height, nose width, mouth width and inter canthal distance. The association between facial measurements with gender and phenotype and across age groups were computed keeping the confidence level at 95%.

**Results:**

Mean age of the children was 72.43±44.2 months with slight male predominance. Thirty-one percent presented with bilateral cleft lip and palate followed by unilateral cleft lip and plate. Total mean facial height, nose width and mouth width were found to be 143.46±21.52mm, 32.24±5.03mm and 33.71±4.38mm respectively. Intercanthal distance was measured to be 31.04±5.99mm. Statistically significant association was observed between gender and facial height, nose width, mouth width and Intercanthal distance.

**Conclusion:**

Facial anthropometric measures done on frontal photographs can be used to identify the facial landmarks in children with non-syndromic cleft lip and palate in low resource stings that may help surgeons in getting better aesthetic outcomes. These landmarks vary between ethnic groups therefore these should be specific to a particular race and ethnicity so as to ensure proper aesthetics and improved quality of life for the children of all nations.

## INTRODUCTION

Orofacial clefts (OFCs) are one of the most frequently diagnosed congenital craniofacial malformations with estimated number of more than 22500 cleft lip and palate births per annum in Pakistan.^1^ These are responsible for major social and psychological burden in the lives of the patients and their families and require a long and multidisciplinary follow-up, including several surgical procedures, orthodontics, and speech therapy.^2^ Facial profile is highly compromised in adult cleft lip and palate patients especially the vertical development of mid face, which is insufficient leading to intrinsic deficiencies of the maxilla. Also, nasal growth is flat and chin is insufficiently developed in the cleft population.^3^ Interocular distance or width, nasal base width, mouth width, lower facial height, nasal length and variable upper lip changes are considered as the main differences affecting facial shape between individuals with cleft lip and palate (CL/P )and unaffected individuals.^4^

Facial appearance influences the quality of life (QoL) of the affected person as it plays a role in the social interactions between individuals and influences a person’s perception of others.^5^ These abnormalities have an important influence on facial attractiveness and psychosocial well-being. CL/P patients are shyer and socially inhibited when compared with non-cleft individuals. They also reported being teased in their childhood and adolescence; and are often stigmatized in social situations.^3^ Comprehensive assessment of a multitude of aspects of CLP is essential.

There is a need to establish a standardization of photographic records in patients with orofacial clefts because the aesthetic evaluation of these patients is an important clinical indicator in facial deformity analysis before surgical and aesthetic management.^6^ It has been reported that Aesthetic outcomes evaluated on two-dimensional (2D) facial photographs and 3D images are equivalent for assessment of some regions of the face, primarily nose, and midface.^7^

Even though the incidence of CLP in Pakistan is high still there is no study documenting facial measurements of children with cleft lip and palate. Normal craniofacial anthropometric values either linear, angular, or proportional are important in diagnostic determination and treatment planning for esthetic and reconstructive dentofacial or craniofacial surgery of patients belonging to different ethnic backgrounds. Therefore, it is important to have a database of normative values for each ethnic group. As universally applied criteria of esthetic attractiveness and proportions may be misleading, due to ethnic variations, the present study was conducted to study the facial morphology in children with cleft lip and palate by applying numerical facial analysis on photographs that may help to evaluate treatment outcomes as well.

## METHODS

This descriptive study was conducted from March 2020 to July 2020 in the Department of oral pathology and histopathology, University of Health Sciences, and Lahore. A total of 104 of both gender with age range from three months to thirteen years were recruited from Cleft Lip and Palate (CLAP) Hospital, Lahore. A written informed consent was taken from their parent or guardians. Demographic data including age, gender and family history were also noted. Children with any type of non-syndromic orofacial clefts were included while children with secondary cleft lip and palate, previous facial surgery or surgical scars on the lip and nose were excluded. Standardized facial photographs from 104 children were taken.

### Ethical Approval:

This study was approved by the institutional ethical committee wide letter # UHS/REG-20/ERC/146. (Date January 15, 2020)

### Photographs:

Facial photographs were taken to determine measurements of facial landmarks. To obtain the photographs individuals sat and kept a normal posture, with both arms free along the body. Background of the pictures was blue. We took the pictures at different angles before and after surgery and at follow up but in current study measurements on pictures before surgery were recorded.

**Fig.1:**
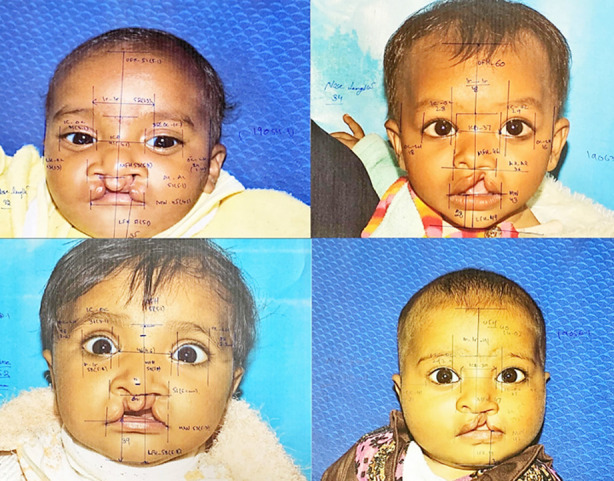
Lands Marks for facial Measurements.

The anthropometrical landmarks were identified, and five measurements were taken including total facial height from Trichion (Tr) to Menton (Me), upper facial height from Trichion (Tr) to Glabella (Gb), middle facial height Glabella (Gb) to Subnasale (Sn) and lower facial height Subnasale (Sn) to Menton (Me) (Fig.1).

### Facial Measurements:

Facial measurements were taken as follows:

### The middle fifth of the face (ICD):

The middle part of the face that is delineated by the inner canthus of the right and the left eyes and should be coincident with the alare of the nose.

### The medial two-fifths of the face (IC-OC):

The medial parts of the face that is delineated by the inner and the lateral canthus of the eyes.

### The lateral two-fifths of the face (OC-LH):

The lateral parts of the face that is delineated by the outer canthus of the eyes and the lateral helix of the ears at the most posterior point on the outer rim of the ear.

***Binocular width (ex-ex):***

### Interalar width (Al-Al):

The distance between the two alare points of the nose.

### Mouth width (Ch-Ch) Cheilion (Ch):

The point located on each labial commissure. The distance between the two angles of the mouth.

### Statistical Analysis:

Statistical analysis was done by using SPSS (version 26) to analyze the data**.** Age of children and all facial measurements are presented as Median and Interquartile range. Mean ± SD was also taken so that the results can be compared with other studies. Gender, family history and cleft type are presented as frequencies and percentages. Mann–Whitney U test was applied to compare the median of facial measurements within gender and cleft phenotypes. Kruskal Wallis test was used to determine association for UFH, MFH, LFH, ICD, IC-OC (for right and left side) and OC-LH (right and left side) across different age groups. For all analysis *p*-value of less than 0.05 was taken as statistically significant keeping the confidence level at 95%.

## RESULTS

**Fig.2:**
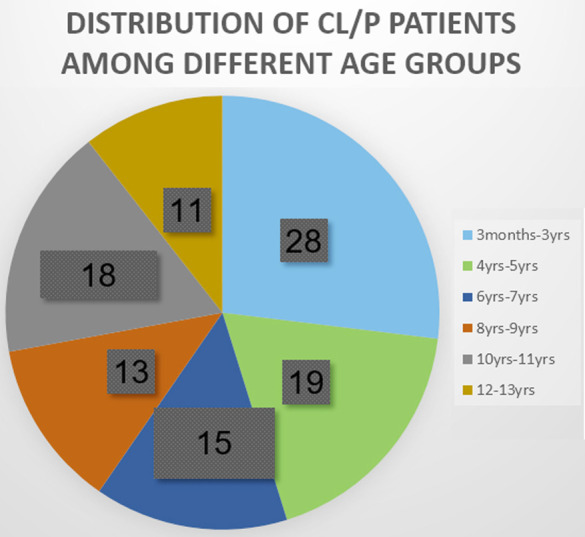
CL/P distribution according to age group (percentage rounded off to nearest zero).

There were 52 males and an equal number of females in 104 children with NSCL&P. Mean age was found to be 72.3±44 months with an age range of three months to thirteen years. Positive family history was recorded in 18 cases only. Regarding age group distribution, most of the children were between the ages of three months to three years as shown in Fig.2. Among 104 children, 57(55%) presented with bilateral cleft lip and palate followed by isolated cleft lip and palate in 27(26%) and 20(19%) children respectively. Regarding laterality, bilateral involvement was seen in 32(38%) children while unilateral involvement affecting right or left side was noted in 22(26%) and 31(36.4%) children. And there were 20 cases of isolated cleft palate in which laterality was not determined.

Mean ± SD and median±IQR of total facial height (TFH) in the study population was found to be 120.7 ± 15.26mm to 174.54±4.2mm and 114.0(111.0-17.0)mm to 174 (172-179) mm in all children aged from three months to thirteen years respectively. Dividing the children into six age groups, there were differences in the Median and IQH among the groups as shown in Table-I.

**Table-I:** Facial Heights, nasal width and mouth width of children with cleft lip and palate within different age groups (mm).

Age group in months (Years)		Total facial height(mm)	Upper facial height(mm)	Middle facial height(mm)	Lower facial height(mm)	Nose Width(mm)	Mouth width(mm)
3-36(0-3 years)	Mean	120.7±15.26	41.32±6.7	39.07±4.68	40.46±5.69	26.07±3.8	28.35±3.3
	Median	114.0 IQ(111.0-127.00)	40.0 IQ(39-43)	37.5(36.0-42.0)	39.5 IQ(36.0-44.0)	24.5 IQ(23.0-27.0)	27.0 IQ(26.0-30.75)
37-60(4-5 years)	Mean	130.89± 9.8	45.26±4.05	42.31±5.11	43.31±5.2	30.8±1.82	33.05±1.77
	Median	127.0 IQ (124-136)	46.0 IQ(43-48)	41 IQ(38-47)	43 IQ(39-46)	31 IQ(29-32)	33 IQ(32-34)
61-84(6-7 years)	Mean	145.8±7.51	48.2±2.14	46.93±3.19	50.60±2.79	33.28±1.4	34.0±1.13
	Median	144 IQ(140-153))	48 IQ(47-50)	46 IQ(44-50)	51 IQ(49-53)	33 IQ(32-34)	34 IQ(33-35)
85-108(8-9 years)	Mean	155.69±11.91	52.5±4.03	51.15±5.24	52.0±5.21	35.00±2.27	35.61±1.2
	Median	157 IQ(152-163)	54 IQ(51-54)	53 IQ(48-55)	53 IQ(49-56)	36 IQ(34-36)	36 IQ(35-36)
109-121(10-11 years)	Mean	162.0±4.15	53.27±1.44	53.83±1.94	55.4±2.35	36.44±1.04	37.67±1.97
	Median	163 IQ(158-165)	53.5 IQ(52-54.25)	54 IQ(53-55)	55 IQ(54.7-57)	36.5 IQ(35.7-37)	38.5 IQ(35-39)
122-156(12-13 years)	Mean	174.54±4.2	56.72±1.3	58.0±1.6	59.63±1.28	38.81±.98	39.27±1.42
Total	Median	174 IQ(172-179)	56 IQ(56-58)	58 IQ(57-59)	60 IQ (59-60)	39 IQ(38-40)	39 IQ(39-40)
	Mean	143.43±21.4	48.13±6.84	46.86±7.7	48.5±8.0	32.25±5.00	33.7±4.36
	Median	144 IQ(124.2-161.75)	49 IQ(43-54)	47 IQ(40-54)	49 IQ(42-55)	33 IQ(28-36)	34 IQ(31-36.7)

Mann-Whitney U test was applied to observe the association of facial measurements with cleft lip and palate type and laterality. Statistically, no significant difference of facial measurements was seen among different types of cleft lip and palate.

The median of distance between inner and outer canthus of right side was found to be 19.54 ± 3.7mm and 18mm with IQ range from 17.25 to 22mm in children of Group-1. The mean and median of the distance between outer canthus of the eyes and the lateral helix of the ears on both right and left side were found comparable as shown in Table-II.

**Table-II:** Inner canthal distance, inner outer canthus distance, and outer canthus lateral helix measurements within age groups.

Age Group Months (Years)		Inner Canthus Distance (mm)	Inner-outer canthus distance (left) mm	Inner-outer canthus distance (Right) mm	Outer Canthus Lateral Helix (Right) mm	Outer Canthus Lateral Helix (Left) mm
3-36(0-3)	Mean	23.7143±3.8	19.17±3.7	19.54±3.7	21.71± 2.27	21.82±1.92
	Median	23IQ (22-24)	17.5IQ (16.25-21.0)	18IQ (17.25-22)	21IQ (20-22)	21IQ (21-22)
37-60(4-5)	Mean	28.89±2.60	22.52±2.01	22.94±1.8	24.42±1.12	24.2±1.65
	Median	29IQ (26-31)	22IQ (21-24)	23IQ (22-24)	24IQ (24-25)	24IQ (23-26)
61-84(6-7)	Mean	32.0±1.96	26.33±1.58	26.13±1.45	27.00±0.92	26.6±1.17
	Median	32IQ (31-34)	27IQ (25-27)	26IQ (25-27)	27IQ (26-28)	27IQ (26-27)
85-108(8-9)	Mean	34.15±2.07	27.07±1.65	27.69±1.49	27.92±1.25	27.46±1.33
	Median	35IQ (32-35)	27IQ (26-28)	28IQ (27-29)	28IQ (27-29)	28IQ (27-28)
109-121(10-11)	Mean	36.6±.97	28.16±1.94	28.44±1.50	29.61±1.03	28.83±1.38
	Median	37IQ (36-37.25)	28IQ (27.75-30)	29IQ (28-29)	29.5IQ (29-30.25)	29IQ (28-30)
122--156(12-13)	Mean	39.27±1.10	31.18±2.9	31.72±3.1	31.00±1.34	30.27±1.55
	Median	39IQ(38-40)	30IQ(30-31)	31IQ(30-32)	31IQ(30-32)	30IQ(29-32)
Total	Mean	31.04±5.9	24.63±4.83	24.9±4.7	26.09±3.6	25.7±3.4
	Median	32IQ(26-36)	26IQ (21-28)	26IQ(22-29)	26IQ(23-28)	27IQ(23-29)

Facial measurements including facial heights, ocular measurements and mouth and nasal width showed a significant association with age groups by applying Kruskal-Wallis test. Table-III shows a difference in means and medians among females and males. Applying Kruskal-Wallis for comparing median revealed this difference statistically significant.

**Table-III:** Association of facial measurements with gender.

Facial Parameters	Gender	Mean ±SD(mm)	P-value*	Median(mm)	P-value*
Total Facial Height(TFH)	M	147.78±21.6	.037	156.5 IQ(126.25-165.0)	0.036
	F	139.0±20.4		138 IQ(124-155)	
Upper Facial Height (UFH)	M	49.3±6.9	.076	52.0 IQ(45.0-54.75)	0.032
	F	46.9±6.61		47 IQ(41-52)	
Middle Facial Height (MFH)	M	46.9±8.09	.045	51.0 IQ(40.25-55.0)	0.039
	F	48.3±7.16		44.5IQ(39.2-50.7)	
Lower Facial Height(LFH)	M	50.25±7.9	.027	53.0 IQ(43.0-56.7)	0.023
	F	46.7±7.85		46.0 IQ(40.0-54.0)	
ICD (InterCanthal Distance)	M	32.3±5.9	.024	34.5 IQ(26.5-37.0)	0.011
	F	29.73±5.7		31.0 IQ (26.0-34.0)	
Inner-Outer Canthus Distance ICD-OCD(Right)	M	25.90±4.26	.050	28.0 IQ(23.0-29.0)	0.017
	F	24.07±5.10		24.0 IQ(20.25-28.0)	
Outer Canthus-Lateral Helix (OC-LH(Right)	M	26.88±3.52	.026	28.0 IQ(24.0-30.0)	0.015
	F	25.3±3.60		26.0 IQ(22.2-28.0)	
Inner-Outer Canthus distance (ICD-OCD(Left)	M	25.59±4.44	.042	27.0 IQ(22.0-29.0)	0.012
	F	23.67±5.05		24.0 IQ(20.25-27.0)	
Outer Canthus-Lateral Helix (OC-LH (Left)	M	26.46±3.37	.036	27.00 IQ(23.25-29.0)	0.024
	F	25.07±3.25		25.5 IQ(22.0-27.7)	
Nose Alae distance (Al-Al)	M	33.34±4.80	.025	35.0 IQ(29.25-37.0)	0.013
	F	31.15±5.00		32.0 IQ(28.0-35.0)	
Distance between angles of mouth (CH-CH)	M	34.71±4.2	.018	35.5 IQ(32.0-38.0)	0.009
	F	32.69±4.31		33.5IQ(31.0-35.7)	

* Independent T test and Kruskal-Wallis test: Association between gender and facial measurements, P< 0.05 was considered as significant.

## DISCUSSION

Facial morphological measurements show variations between racial and ethnic group with changes in different ages. Face and cranium basic measurements help in classifying, diagnosing and treating craniofacial anomalies in an objective manner. To date a number of qualitative and quantitative measurements methods have been used to determine the facial soft tissue appearance of patients with CL/P.^8^ As the patients with CL/P show different facial morphology and growth patterns than unaffected individuals^9^ therefore it is essential to have this data available in all populations. This is the first study of these measurements from children with CL/P from Pakistan.

In cleft patients, facial photographs and lateral cephalograms are used mainly to determine the measurements.^10^ Nagy and his colleague evaluated nasal form and symmetry in cleft patients by using photographs. They found photographs as an appropriate source for nasal analysis while comparing the results of different surgical techniques.^11^

The current study observed mean age of participants to be 72.43±44.2 months (6years) with approximately equal gender distribution. Jamilian A et al. found mean age as 12.3 ± 4 years in males and 12.6 ± 3.9 years in females among 201 cleft patients with male predominance.^12^ Another similar study found more males affected while comparing to females and cleft palate being predominant among all types of clefts.^13^ Among the type of clefts, one investigation found 42% of total patients belonging to cleft palate only category contrary to present findings where combined cleft lip and palate is the most common type. The reason for these variations can be ethnicity, different genetic makeup, habits and environmental factors as all these are associated with the variations seen in age, gender and cleft type.^14^

### Facial height:

The current study found mean facial height as 143.4±21.5mm in cleft lip and palate patients aged from three months to 13 Years. Othman SA reported lower facial height as 60.62 mm, upper face height as 46.41 ± 4.48 and the total facial height as 105.55 ± 8.99 mm in 7-12 years olds Chinese children with cleft lip and palate.^15^ The difference of 7.12mm, 6.09mm, and 53.5mm was noted while comparing the UFH, LFH and TFH with our study respectively. Similarly, Zreaqat et al. and his fellows found total facial height as 110.7 ± 5.10mm in ULCL/P in Malay children aged 8-10 years old.^16^ The reason behind the differences of 53.5mm and 51.3mm in TFH was the selection of different landmarks by Othman et al. and Zreaqat et al. while in our study it was taken from Trichion to Menton.^15^,^16^

Jahanbin A et al. took facial photographs of 662 newborns to 12 years old Iranian girls with normal facial morphology and determined facial height from Nasion to Gnathion which was 58.39± 11.11mm (in less than one year) and 100.31±5.67mm (11years).^17^ Bossle R et al. conducted a study on 93 children aged from three to five years old with normal morphology and found mean facial height of 100.9±5.7mm in males and 97.6±5 mm in females.^18^ While the current study reported mean facial height as 130.89± 9.8mm in children of 3-5 years age group with cleft lip and palate thus showing a difference in facial height in children with normal morphology and having cleft lip and palate.

Another study reported total facial height of 114.2± 5.13mm in normal 8 to 10 years old children of ethnic origin of Malaya.^16^ While CL/P children of the same age showed FH from 155.69±11.91mm to 162.0±4.15mm in the present study. In seven years to eleven years old Brazilian children facial height was found as 170.9mm in 11 years olds (Cattoni DM et al.,2009) while it was found to be 162.0±4.15 mm in 11 years old children in the current study.^19^ The differences in TFH can be because of different ethnicities and the selected landmarks as the other studied measured TFH from nasion to gnathion while in our study it was taken from Trichion to Menton.

### Intercanthal Width:

The current study reported mean intercanthal width as 31.04±5.9mm in children from three months to 13 years old with orofacial clefts. Othman SA found mean bi-ocular and intercanthal width as 88.71 ±3.87mm and 36.01±2.07mm in cleft children while these measurements were found as 87.77 ±4.86mm and 35.20 ±3.42mm in normal children of 7-12 years old of age group in the same study.^15^ Present study reported intercanthal width as 36.63±1.38 mm in CL/P children of same age group (7-12 years), with a difference of only 0.635mm from the previously reported study. Zreaqat et al. and Yamada et al. reported narrow intercanthal width in children affected with UCLP than their normal counterparts, However, in both cases these findings were not significant as their mean difference was too small.^16^,^20^ Zreaqat M compared the intercanthal distance in cleft lip and palate children and normal children of same age group (9.4 years mean age) and found the difference of only 0.9 mm. It can be implied from these studies that these measurements of CL/P in local population will correspond to the normal population of the country.^16^ A possible explanation for the discrepancy between these studies might be variations in populations.^21^

Jahanbin A et al. measured Intercanthal width as a distance between endocanthion (en) the soft tissue point located at the inner commissure of each eye fissure in 0 to 11 years old normal Iranian children and reported 23.18 ±2.96mm to 28.68 ±2.62mm.^17^ The current study found Intercanthal width from 23.7143±3.8mm to 36.6±.97mm in CL/P children of same age group by using similar landmarks and showed a difference with increasing age as the distance between eyes increases with advancing age resulting in increased intercanthal width.

Mahdi E et al. performed his study on 564, 4-11 years old boys of Kurmanj ethnic origin from Shirvan, with normal face patterns and reported intercanthal width from 26.3±1.8mm(four years) to 28.6±2.6mm(11 years), with a difference of approximately 3.0mm and 8.0mm for four years to 11 years while compared to CL/P children of the same ages.^22^ Rushil and colleagues reported Intercanthal width as 24.4mm to 25.9mm in normal children up to one years aged Caucasian children.^23^ The present study showed a difference of few millimeters in Intercanthal width of CL/P children belonging to same age group. Intercanthal distance along with other landmarks can be used as an important parameter for two-dimensional reconstruction of face.^24^

Right and left ocular width was reported as 27.63 ±1.84mm and 27.7 ±1.88 mm in cleft lip and palate patients of 7-12 years old Chinese children respectively by Othman SA et al.^15^ He also found right ocular width as 27.42 ±1.8 mm and left ocular width as 27.38 ±1.7 mm in normal children.^15^ The present study reported these measurements with a difference of only 0.43mm and 0.08mm for right and left ocular width respectively.

Mohammed DR et al. reported OC-LH (right) as 35.7mm and OC-LH (left) as 38.6mm in adults with normal occlusion and facial morphology while in current study OC-LH (right) and OC-LH (left) were found as 26.09±3.6 mm and 25.7±3.4mm in children from 3 months to 13 years old children with orofacial clefts.^25^ These findings show the minimal differences in measurements between cleft patients and normal adults. One of the reasons behind this difference can also be the age of the participants

### AL-AL length:

Othman SA wider alar width in patients with cleft lip and palate but did not find any significant association with normal alar base width.^15^ However, Zreaqat et al. observed a significantly wider alar base width in cleft children of Malay ethnicity with a mean difference of 2.89 mm.^16^ Jahanbin A et al. found alar width from 24.33 ±2.60mm in children less than one year to 32.90 ±2.15mm in 11years old children with a difference of 2.0 to 4.0mm while compared to current study 26.07±3.81 and 36.4±1.4mm respectively.^17^ Mahdi E et al. and Ogodescu E et al. found a difference of few millimeters in nose width in Romanian and Iranian children with normal morphology of aged 3.5 to14 years.^22^,^26^

### Mouth Width:

Zreaqat M et al. reported 48.9 ±3.69mm mouth width in children having orofacial clefts while in children with normal morphology it was found as 50.3±5.72mm with no statistical significance.^16^ The current study reported 28.35 ±1.82mm to 39.27±1.42mm mouth width in children with orofacial clefts from three months to 13years. Jahanbin A measured mouth width in normal children aged from less than one year (27.02±3.57mm) to 11 years (44.10 ±3.37mm).^17^ Ogodescu E et al. reported mouth width as 35.93 ± 3.43mm in 3.5-5years old children and 44.14 ± 3.93mm in 11.5-13 years old normal Romanian children.^26^ Esmaeilzadeh Mahdi found 35.1±2.5 mm. to 44.1 ± 3,5 mm mouth width in children aged from 4-11 years old.^22^ Mouth width differs among in all above mentioned studies ranging from 01mm to 5.0 mm maximally with increasing age.

Though a number of assessment techniques like 2-dimensional (2D) photographs, 3-dimensional images, videography and direct clinical examination are used to evaluate facial symmetry and nasolabial aesthetics yet no internationally accepted standardized rating method for the aesthetic evaluation of patients with cleft after cleft repair is recognized.^27^ Cleft lip and palate patients still lack the reference values in infants therefore craniofacial anthropometry can be used as in many other areas.^28^

### Association between facial measurements and gender:

The current study showed a significant association between certain facial measurements with gender; however, Othman reported no significant gender differences (p= 0.851).^15^ Despite the numerous surgical procedures that aim to improve facial esthetics, BCL/P patients are usually unsatisfied with the appearance of their upper lip and nose. In addition, boys were more dissatisfied than girls on this issue. From the patient’s perspective, the esthetic concerns are greater than the functional concerns.^29^

Pre-operative severity of clefts and pre- operative measures determine post-operative appearance. Different anthropometric measures including angle of columellae, nasal width, and lateral lip height are important and can be employed assessing longitudinal treatment of the unilateral cleft lip nasal deformity and must be included as objective measures. So, the cleft lip and palate surgery is performed not only to restore normal functions like eating, respiration and speech^30^ but it also plays an important role in restoring the aesthetics for which proper facial measurements are considered significant.

### Limitations:

This is a single center study with small sample size. There are certain resources constrains like 3D images. Also, more facial measurements can be included in order to evaluate facial proportions and symmetry that may aid in treatment planning and aesthetic outcomes.

## CONCLUSION

There facial measurements of children with cleft lip and palate across age groups is specific to the stage of development of the study participants. The difference is not related to cleft phenotype however is gender specific. These dimensions highlight the importance of determining proper facial measurements in cleft lip and palate patients of varying age groups to be able to align the measurements with that of normal children of the same age groups before commencing any surgical procedure in order to get the best aesthetic outcomes for these children. This also ensures better psychological outcomes for these individuals thus having a profound effect on their self esteem and quality of life.
